# An Analytical Framework for the IEEE 802.15.4 MAC Layer Protocol under Periodic Traffic

**DOI:** 10.3390/s20123350

**Published:** 2020-06-12

**Authors:** Yipeng Wang, Wei Yang, Ruisong Han, Linsen Xu, Haojiang Zhao

**Affiliations:** 1 School of Electronic and Information Engineering, Beijing Jiaotong University, Beijing 100044, China; 14111029@bjtu.edu.cn (Y.W.); linsenx@bjtu.edu.cn (L.X.); 11111017@bjtu.edu.cn (H.Z.); 2 Telecommunications Software & Systems Group (TSSG), Waterford Institute of Technology, X91 P20H Waterford, Ireland; rhan@tssg.org

**Keywords:** IEEE 802.15.4, periodic traffic, CSMA/CA, analytical framework, network throughput, energy consumption

## Abstract

As the reference communication standard of wireless sensor networks (WSNs), the IEEE 802.15.4 standard has been adopted in various WSN-based applications. In many of these applications, one of the most common traffic pattern types is a periodic traffic patterns, however, the majority of existing analytical models target either saturated or unsaturated network traffic patterns. Furthermore, few of them can be directly extended to the periodic traffic scenario, since periodic traffic brings unstable load status to sensor nodes. To better characterize the WSNs with periodic traffic, we propose an accurate and scalable analytical framework for the IEEE 802.15.4 MAC protocol. By formulating the relationship between clear channel assessment (CCA) and its successful probability from the perspective of channel state and node state, single node’s behavior and whole network’s performance under different network scales and traffic loads can be derived. Extensive simulations are conducted to validate the proposed framework in terms of both local statistics and overall statistics, and the results show that the model can represent the actual behavior and the real performance of both single node and whole network. Besides, as the simplified version of double CCAs mode (DS mode), single CCA mode (SS mode), is also analyzed with simple modifications on the proposed analytical framework. Combining the analytical framework with simulation results, the applicable network scenarios of two modes are also demonstrated respectively. Finally, an approximate distribution of one data packet’s backoff duration is proposed. With this approximate distribution, a conservative estimation of data packet’s average transmission latency in networks with given configurations can be easily carried out.

## 1. Introduction

Wireless sensor networks (WSNs) have penetrated various kinds of applications, ranging from habitat monitoring to industrial process control, for their advantages of low deployment costs, ease of installation, maintenance and reconfiguration, and the inherent intelligent-processing capability over traditional wired devices [1]. The IEEE 802.15.4 standard is the reference communication technology for WSNs. It specifies the protocols of the physical layer and medium access control (MAC) layer for WSN devices [2].

According to the standard, the MAC layer is responsible for scheduling nodes’ channel access operations, which greatly affects their data transmission probability. The MAC layer of IEEE 802.15.4 standard provides several channel access mechanisms to cope with different communication requirements. Among them, the slotted carrier sense multiple access with collision avoidance (slotted CSMA/CA) protocol is the most widely used one. To reduce the evaluation efforts of IEEE 802.15.4 based WSNs, many analytical models for slotted CSMA/CA protocol have been proposed. According to the assumed traffic pattern, the majority of them can be categorized as either saturated traffic pattern adapted model or unsaturated traffic pattern adapted model [3,4,5,6,7,8,9,10,11]. With saturated traffic pattern, nodes always have data packets to be transmitted. This case can be used to model the performance of WSNs in which several sensors want to send data at the same moment, e.g., when a critical event is detected. Whereas, in WSNs with unsaturated traffic pattern, nodes are always assumed to wait for a period of time before new data packets arrive, in real-life scenarios, many applications are required to send data periodically, resulting in the periodic traffic pattern. For example, in WSNs that support advanced metering infrastructure (AMI) applications, sensors are usually configured to periodically send application-specific data (such as electricity consumption) to a base station collector for system monitoring and analysis [12,13]. Another example is the transmission line monitoring applications. In the electricity distribution part of smart grids, sensor nodes are used to metering the current, voltage and temperature of transmission line periodically for the sake of a reliable power system [13]. It can be expected that each node’s load status will probably switch between saturated and unsaturated with periodic traffic. To accurately characterize the MAC behavior of nodes and the performance of WSNs with periodic traffic, we propose a competent analytical framework of the slotted CSMA/CA protocol. The framework is unfolded from two perspectives, channel state and node state. By formulating the relationship between the probability of performing clear channel assessment (CCA) and its successful probability from both perspectives, the performance statistics of single node and whole network under different scales and traffic loads are carried out. Our main contributions can be summarized as follows:

(1) Different from assuming single CCA mode (SS mode) as previous works do, this paper proposes a Markov chain-based channel state model which supports the analysis of networks adopting double CCA mode (DS mode). In the proposed channel model, we pay more attention to the operation of the 1st CCA (CCA1). The relationship between the occurrence of CCA1 in a channel and its successful probability is formulated.
(2) Contrary to approximating the distribution of backoff duration with continuous probability analysis, we consider the duration of backoff period as a discrete time signal and analyze it with signal processing approach. With the assistance of a discrete Fourier transform (DFT), the distribution of a single node’s backoff duration is obtained. Based on the distribution of a single node’s backoff duration, single node’s CCA1 performing probability and its successful probability are formulated. Besides, the statistics of single node’s load status are also determined.
(3) Combining the analysis of channel state and node state, an analytical framework of IEEE 802.15.4 MAC layer protocol is proposed. With a given network scale, data packet’s inter-arrival time and channel access parameters, the performance statistics of a single node and the whole network can be estimated.
(4) With simple modifications, the proposed analytical framework can be modified to be compatible with SS mode. By comparing DS mode and SS mode in different network scenarios, the paper demonstrates applicable network scenarios of the two modes, respectively.
(5) By approximating the distribution of one data packet’s backoff duration as a normal distribution, we come up with a method of estimating data packet’s average transmission latency in networks with given configurations. Simulation results show that the proposed method provides a conservative and reliable estimation on network’s average transmission latency and can be used to judge whether or not a given network’s average transmission latency is bounded by packet inter-arrival time.

The remainder of the paper is organized as follows: Related work is given in Section 2. Section 3 briefly introduces theslotted CSMA/CA algorithm. Section 4 presents the analytical frameworks. In Section 5, critical issues concerning data transport performance and energy efficiency are proposed and analytically characterized. In Section 6, rational analysis and performance evaluation of the proposed analytical model and critical issues optimizations are presented. Section 7 provides our concluding remarks and discussions of the results.

## 2. Related Works

In recent years, the IEEE 802.15.4 MAC protocol has been thoroughly investigated. For more accurate network performance evaluation and more efficient network performance optimization, many researchers work on modeling the behavior of the MAC protocol. In general, the behavior of the IEEE 802.15.4 MAC protocol is usually modeled from two perspectives, i.e., node state and channel state. From both perspectives, the Markov chain-based model is often adopted.

Inspired by Bianchi’s work [14], a number of analytical models characterize the behavior of nodes with the Markov chain-based model [3,4,5,6,7]. They usually divide and categorize the behaviors of single node into different states. By obtaining the transition probability among different states, the behavior of single node can be presented. However, the majority of them assume the traffic pattern of nodes to be consistently saturated or unsaturated. The reason for this is that, with consistent load status, the steady-state probability of different node state is available. The model in [15] assumes a saturated traffic pattern at first and then is extended to accommodate an unsaturated periodic traffic pattern. Targeting unsaturated traffic scenarios, many existing Markov chain-based node state models assume that the traffic pattern of a single node follows either a Poisson distribution [8,9,11,16] or a uniform distribution [10], while with the periodic traffic pattern, the load status of a node cannot keep consistent and the steady state of the corresponding Markov chain based model is not available [17]. Hence, leveraging the Markov chain-based model to characterize the node’s behavior is no longer applicable under the periodic traffic pattern. The authors propose an adjacent slot-pair Markov chain model to analyze the transition behavior of the channel state. The proposed model is rather simple yet heuristic. Several analytical models and channel access optimization algorithms are inspired by it. However, although the authors in [18] claim the proposed channel state model is compatible with the nodes in DS mode, we will show in this paper that its applicability to DS mode still needs to be improved. The research in [19] characterizes the channel state change with the Markov chain-based model.

Periodic traffic brings unstable load status to sensor nodes, which causes the variation of MAC statistics over time. This is the reason that the majority of existing stationary Markov chain model-based node state analyses cannot be directly extended from either saturated or unsaturated traffic scenarios to the periodic traffic scenario. To our best knowledge, few analytical IEEE 802.15.4 MAC models have been proposed for the periodic traffic scenario.

The research in [19] addresses a kind of periodic traffic scenarios that each node has a packet to transmit at the beginning of any contention period. The number of nodes with different states and the variation of channel states are covered in a non-stationary 3-D Markov chain model. As an extension of [19,20], proposes a 4-D Markov chain-based model. However, both models assume nodes in SS mode instead of DS mode. The model proposed in [17] accounts for DS mode. The model focuses on the time-varying feature of the MAC statistics under periodic traffic, while, similar to [19], the model in [17] assumes that each node only has one data packet to be transmitted during each contention period. However, the time interval of monitoring results or measurement reports is mainly determined by the application requirement rather than the duration of the contention period.

## 3. The Slotted CSMA-CA Protocol

In this section, one of the IEEE 802.15.4 MAC protocols, the slotted CSMA-CA protocol targeting beacon-enabled WSNs is introduced. Besides, the considered network scenarios and model assumptions are also indicated. In the beacon-enabled WSNs, the network is composed of network coordinators and general sensor nodes. The network coordinator periodically sends beacon frames for network synchronization. The time interval between two consecutive beacon frames is called the beacon interval (BI). Every sensor node generates data packets and contends to send them after receiving beacon frames. Table 1 presents the notations and definitions of key MAC layer parameters, and lists the parameters defining the network scenario.

**Figure 1:**
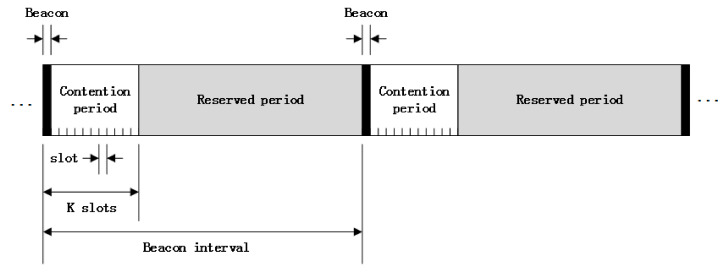
Beacon interval structure.

Figure 1 presents the structure of BI. In beacon-enabled WSNs, continuous time is divided into discrete time slots. The duration of each slot is fixed to 320 μs. The length of BI is decided by the value of the beacon order (*BO*), where 0≤BO≤14. The period of the BI is composed of an active period and an inactive period. The active period is structured as a superframe for data packet transmission. The superframe duration (*SD*) is determined by the value of the superframe order (*SO*), where 0≤SO≤BO≤14. Within one superframe, the first slot is allocated for the beacon frame and the rest of the time slots are divided into contention access period (CAP) and contention free period (CFP).

**Figure 2:**
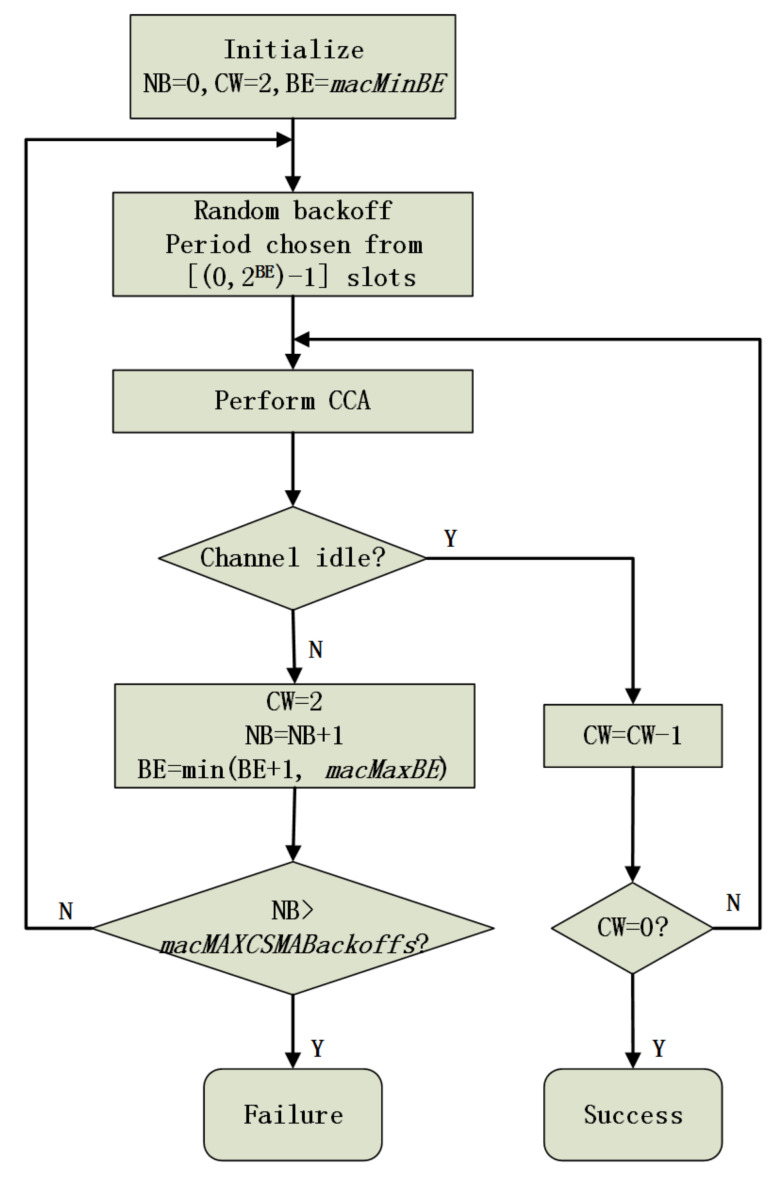
Slotted CSMA/CA protocol.

As is shown in Figure 2, according to slotted CSMA-CA, three parameters are initialized when data packets need to be transmitted, i.e., the number of backoff stages (*NB*), the contention window (*CW*), and the backoff exponent (*BE*). Every packet transmission only happens after the channel is identified as idle. The identification of the idle state is accomplished through two consecutive successful CCAs. We term the 1st CCA operation as CCA1 and the 2nd CCA operation as CCA2. Every time before performing CCA1, a sensor node should wait for several time slots. The number of slots is randomly selected from 0 to $2^{BE}-1$. If either CCA fails, then *NB* and *BE* are both increased by 1, and *CW* is reset to 2. The current packet transmission is identified as failed if the value of *NB* reaches its maximum value *macMaxCSMABackoffs*. If the value of *BE* reaches the maximum *macMaxBE*, then the sensor node keeps its value until a successful/failed packet transmission occurs.

In the remainder of this work, we consider a WSN working in the beacon-enabled mode with star topology. One network coordinator at the center and *M* sensor nodes randomly deployed around the network coordinator within the carrier sensing range. A star topology is the most common topology and it may also exist inside clusters in larger-scale 802.15.4 networks. According to the standard, nodes can’t start their channel access attempt until receiving the beacon frame. To eliminate the effect of beacon frame receiving and present the relationship between the arrival of data packets and the channel access attempt more explicit, we assume beacon frames are loss-free.

The delay of signal propagation and frame processing is assumed to be negligible, so that all the nodes can receive beacons and contend for channel access simultaneously. Sensor nodes are assumed to be homogeneous and their MAC protocols are configured with the same parameters. Sensor nodes are assumed to be homogeneous and their MAC protocols are configured with the same parameters. For each sensor node, the inter-arrival time of data packets is assumed to be *T* sequential time slots. Given that all sensor nodes are not synchronized, each sensor node is assumed to start its periodic reporting at a uniformly distributed random time between 0 and *T*. All data packets are of the same length, and the transmission of one data packet takes *L* time slots. ACK frames and the retransmission function are disabled, which means that collided packets will not be retransmitted. Since the slotted CSMA/CA mechanism is defined for channel access during CAP, we only consider the part of contention access period (CAP) in active period. Beacon period and contention-free period (CFP) in active period as well as inactive period are beyond the consideration.

## 4. Analytical Framework

### 4.1. Channel State Analysis

**Figure 3:**
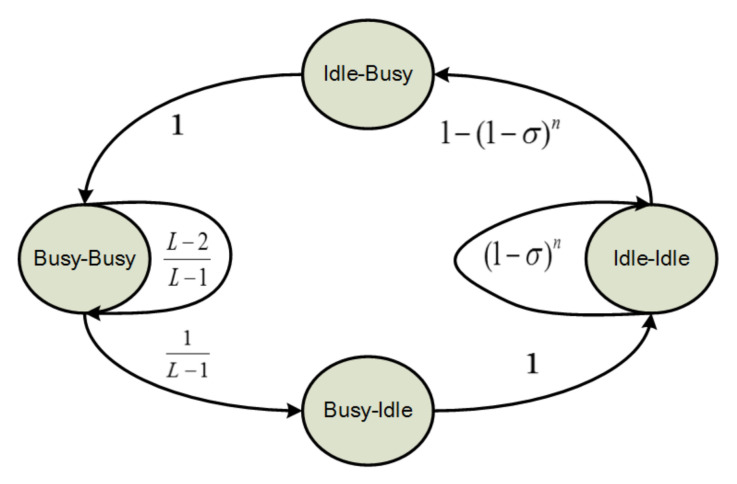
Adjacent-slot-pair-wise channel state model from [19].

The existing Markov chain-based channel state model is shown in Figure 3 [18]. Busy channel state stands for the channel is occupied with packet transmission. Other operations in a channel can be summarized as idle channel state, which includes no operation, CCA1 operation and CCA2 operation. The symbol σ stands for the probability of a single node performing CCA operation in any slot, which includes both CCA1 and CCA2. For channels with nodes in double CCAs mode, the transition from Idle-Idle state to idle-busy state only happens after successful CCA2 operation, so the corresponding transition probability is not $1-{\left(1-\sigma \right)}^{n}$. Besides, according to Figure 3, the channel stays in idle-idle state with probability ${\left(1-\sigma \right)}^{n}$, which doesn’t include the happening of any node performing CCA1 and CCA2 in successive slots.

**Figure 4:**
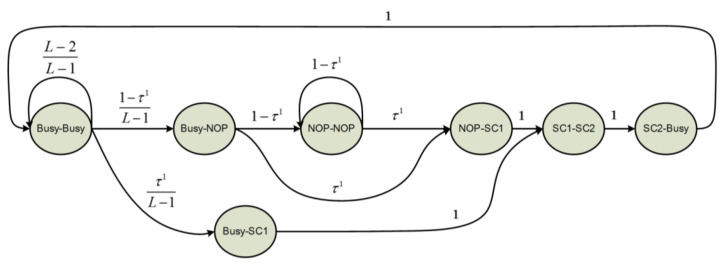
The proposed adjacent-slot-pair-wise channel state model.

In the proposed channel state model. We divide the channel status into four different states, busy state (Busy), successful CCA1 (SC1) state, successful CCA2 (SC2) state, and no operation (NOP) state. The busy state stands for a channel is occupied with data packet transmission. SC1 state stands for one or more successful CCA1 operations are happening in the current slot. Similarly, SC2 state stands for one or more successful CCA2 operations happening, while, if both successful CCA1 and successful CCA2 exist in one slot, the channel should be classified as SC2 state. NOP state represents no channel sense or packet transmission happen in current slot. These four channel states are mutually exclusive. Figure 4 shows the proposed adjacent-slot-pair-wise channel state model.

The symbol $\tau^{1}$ represents the probability of CCA1 operation happens in a slot. According to the state transitions thus modeled, the stationary channel state probabilities are related as follows:

(1) $$\{\begin{array}{l} P_{NN}^{}+P_{NC1_{S}}^{}+P_{C1_{S}C2_{S}}^{}+P_{C2_{S}B}^{}+P_{BI}^{}+P_{BC1_{S}}^{}+P_{BB}^{}=1\\ P_{NN}^{}=(1-\tau_{}^{1})\cdot P_{NN}^{}+(1-\tau_{}^{1})\cdot P_{BN}^{}\\ P_{NC1_{S}}^{}=\tau_{}^{1}\cdot P_{NN}^{}+\tau_{}^{1}\cdot P_{BN}^{}\\ P_{C1_{S}C2_{S}}^{}=P_{NC1_{S}}^{}+P_{BC1_{S}}^{}\\ P_{C2_{S}B}^{}=P_{C1_{S}C2_{S}}^{}\\ P_{BN}^{}=\frac{\left(1-\tau_{}^{1}\right)}{\left(L-1\right)}\cdot P_{BB}^{}\\ P_{BC1_{S}}^{i}=\frac{\tau_{}^{1}}{\left(L-1\right)}\cdot P_{BB}^{}\\ P_{BB}^{}=P_{C2_{S}B}^{}+\frac{\left(L-2\right)}{\left(L-1\right)}P_{BB}^{} \end{array}$$

Solving the above equations yields:

(2) $$\left\{\begin{array}{l} P_{NN}^{}=\frac{{\left(1-\tau_{}^{1}\right)}^{2}}{1+\tau_{}^{1}(1+L)}\\ P_{NC1_{S}}^{}=\frac{\tau_{}^{1}(1-\tau_{}^{1})}{1+\tau_{}^{1}(1+L)}\\ P_{C1_{S}C2_{S}}^{}=\frac{\tau_{}^{1}}{1+\tau_{}^{1}(1+L)}\\ P_{C2_{S}B}^{}=\frac{\tau_{}^{1}}{1+\tau_{}^{1}(1+L)}\\ P_{BN}^{}=\frac{\tau_{}^{1}(1-\tau_{}^{1})}{1+\tau_{}^{1}(1+L)}\\ P_{BC1_{S}}^{}=\frac{{\left(\tau_{}^{1}\right)}^{2}}{1+\tau_{}^{1}(1+L)}\\ P_{BB}^{}=\frac{\left(L-1)(\tau_{}^{1}\right)}{1+\tau_{}^{1}(1+L)} \end{array}\right.$$

As successful carrier sensing is possible only when a channel is not occupied, the probability of successful CCA1 operation $P_{cca1}^{S}$ can be expressed as:

(3) $$P_{cca1}^{S}=1-P_{C2_{S}B}^{}-P_{BB}^{}=\frac{1+\tau_{}^{1}}{1+\tau_{}^{1}(1+L)}.$$

Accordingly, successful CCA2 only happens in neither previous slot nor current slot been occupied:

(4) $$P_{cca2}^{S}=\frac{P_{C1_{S}C2_{S}}^{}+P_{NN}^{}+P_{NC1_{S}}^{}}{1-P_{BN}^{}-P_{BCI_{S}}^{}-P_{BB}^{}}=\frac{1}{1+\tau_{}^{1}}.$$

The probability of successful channel access for each node is:

(5) $$P_{cca}^{S}=P_{cca1}^{S}\cdot P_{cca2}^{S}=\frac{1}{1+\tau_{}^{1}(1+L)}.$$

### 4.2. Node State Analysis

Although the probability of one node’s successful channel access is affected by other nodes’ channel access attempts, the attempt of channel sense is motivated by the need of self-data-packet’s transmission. For sensor nodes in beacon-enabled mode, the operations include backing off, channel sensing, packet transmission, and sleeping. Compared with channel sensing and packet transmission, the length of node’s backoff period may varies a lot. Hence, in this section we formulate the distribution of node’s backoff period firstly. Then, with the consideration of CCA operation and data packet transmission, the distribution of time that a node spends on one data packet is also derived.

#### 4.2.1. The Distribution of Backoff Period

According to the slotted CSMA/CA protocol, the length of the backoff period $\zeta_{i}^{}$ in *i*th backoff stage is a random variable uniformly distributed in the closed interval $\left[0,2^{BE_{i}}-1\right]$. The probability mass function (PMF) of $\zeta_{i}^{}$ is the natural-valued function of an integer variable given by:

(6) $$g_{i}^{}(n):=\left\{\begin{array}{ll} \frac{1}{2^{BE_{i}}} & \mathrm{if}\;n\leq 2^{BE_{i}}-1\\ 0 & \mathrm{if}\;n>2^{BE_{i}}-1 \end{array}\right..$$

If one data packet is transmitted or dropped after *NB* times of backoff, the total number of backoff slots for this data follows the distribution of a sum of *NB* independent uniform random variables with different ranges. The PMF of the sum $\sum_{i=1}^{NB}\zeta_{i}^{}$ is given by the *NB*-fold linear integer convolution:

(7) $$f_{NB}^{}(n):=\left(g_{1}*g_{2}*\cdots *g_{NB}\right)\left(n\right)=\sum_{k_{1}+k_{2}+\cdots +k_{NB}=n}\prod_{j=1}^{NB}g_{j}(k_{j}).$$

For the random variable $\zeta_{i}^{}$, its PMF can be presented as a discrete sequence of taking each value’s probability:

(8) $$g_{i}^{}\left(n\right)=\frac{1}{N_{i}},$$

where $n\in [0,N_{i}-1]$.

The *N*-point Discrete Fourier Transform (DFT) of sequence $g_{i}^{}\left(n\right)$ can be expressed as an *N-N* matrix multiplication as:

(9) $$G_{i}\left(k\right)=g_{i}\left(n\right)\cdot X_{i},$$

where $G_{i}\left(k\right)$ is the DFT of sequence $g_{i}^{}\left(n\right)$. The transformation $X_{i}$ can be defined as a matrix of size N×N:

(10) $$X_{i}=\left[\begin{array}{llll} 1 & 1 & \cdots & 1\\ 1 & x_{i} & \cdots & x_{i}^{N_{i}-1}\\ \vdots & \vdots & \ddots & \vdots \\ 1 & x_{i}^{N_{i}-1} & \cdots & x_{i}^{\left(N_{i}-1\right)\left(N_{i}-1\right)} \end{array}\right],$$

where $x_{i}=e^{-j2\pi /N_{i}}$.

The corresponding Inverse Discrete Fourier Transform (IDFT) is:

(11) $$g_{i}\left(n\right)=\frac{1}{N_{i}}G_{i}\left(k\right)\cdot X_{i}^{-1}.$$

According to the property of DFT, the product of two sequences’ DFT output is the DFT of their circular convolution. With the addition of another random variable $\zeta_{j}^{\prime }$ from $g_{i}^{}\left(n\right)$, this property can be expressed as:

(12) $$g_{i}\left(n\right)⊗g_{j}\left(n\right)=IDFT\left[G_{i}(k)\cdot G_{i}(k)\right].$$

However, as is mentioned earlier, the PMF of the sum of $\zeta_{i}^{\prime }$ and $\zeta_{j}^{\prime }$ is equal to their liner integer convolution. To find out the linear convolution, we first create two new sequences by zero-padding the original sequences to a length of $N_{\left(2\right)}$ points:

(13) $$g_{i}^{\prime }(n)=\left\{\begin{array}{ll} g_{i}^{}(n), & n\in [0,N_{i}-1]\\ 0, & n\in [N_{i},N_{2}-1] \end{array}\right.,$$

(14) $$g_{j}^{\prime }(n)=\left\{\begin{array}{ll} g_{j}^{}(n), & n\in [0,N_{j}-1]\\ 0, & n\in [N_{j},N_{2}-1] \end{array}\right.,$$

According to the theory of discrete-time signal processing [21], the circular convolution of two sequences can be converted to a linear convolution with the help of zero-padding. For example, there are two sequences *x1(n)* and *x2(n)* of length *N1* and *N2*, respectively. Their circular convolution can be converted to the linear convolution of their zero-padded sequences *x1’(n)* and *x2’(n)*. And the lengths of both *x1’(n)* and *x2’(n)* are *N1 + N2−1*. Accordingly, the value of $N_{\left(2\right)}$ in function (13) and function (14) should be $2^{BE_{i}}+2^{BE_{j}}-1$ and the following equation holds:

(15) $$g_{i}(n)*g_{j}(n)=IDFT\left[G_{i}^{\prime }(k)\cdot G_{j}^{\prime }(k)\right],$$

where $G_{i}^{\prime }\left(k\right)$ and $G_{j}^{\prime }\left(k\right)$ are the DFT of sequence $g_{i}^{\prime }\left(n\right)$ and $g_{j}^{\prime }\left(n\right)$, respectively. Hence, the PMF of $\zeta_{i}^{\prime }+\zeta_{j}^{\prime }$ can be expressed as:

(16) $$f_{\left(2\right)}\left(n\right)=\frac{1}{N_{\left(2\right)}}G_{\left(2\right)}\left(k\right)\cdot X_{\left(2\right)}{}^{-1},$$

where $G_{\left(2\right)}\left(k\right)=G_{i}^{\prime }\left(k\right)\cdot G_{j}^{\prime }\left(k\right)$. And the term $x_{\left(2\right)}$ in matrix $X_{\left(2\right)}$ can be expressed as $x_{\left(2\right)}=e^{-j2\pi /N_{\left(2\right)}}$. By using a mathematical inductive method, the PMF of $\sum_{i=1}^{NB}\zeta_{i}^{}$ can be derived:

(17) $$f_{\left(NB\right)}^{}(n)=\frac{1}{N_{\left(NB\right)}}G_{\left(NB\right)}\left(k\right)\cdot X_{\left(NB\right)}{}^{-1},$$

where $N_{\left(NB\right)}=\sum_{i=1}^{NB}2^{BE_{i}}$, $G_{\left(NB\right)}\left(k\right)=\prod_{i=1}^{NB}G_{i}^{\prime }\left(k\right)$ and $x_{\left(NB\right)}=e^{-j2\pi /N_{\left(NB\right)}}$.

Considering the probability of successful channel access, we can obtain the distribution of time that a sensor node spends on backoff for one data packet as:

(18) $$f(n)=\sum_{i=1}^{NB-1}P_{cca}^{S}\cdot {\left(1-P_{cca}^{S}\right)}^{i-1}\cdot f_{\left(i\right)}(n)+{\left(1-P_{cca}^{S}\right)}^{NB-1}\cdot f_{\left(NB\right)}(n),$$

where $n\leq N_{\left(NB\right)}-1$.

The cumulative distribution function (CDF) of backoff duration is:

(19) $$F(n)=\sum_{i=0}^{n}f(i),$$

For a given set of backoff parameters, the value of $N_{\left(NB\right)}$ and $x_{\left(NB\right)}$ are specified. Accordingly, the DFT sequence $G_{\left(NB\right)}(k)$ can be determined. Hence, the distribution of time for backoff operation is only related to the probability of successful channel access $P_{cca}^{S}$:

(20) $$f(n)=\alpha \left(n,P_{cca}^{S}\right),$$

Similarly, the CDF of backoff duration can be expressed as:

(21) $$F(n)=\beta \left(n,P_{cca}^{S}\right),$$

#### 4.2.2. The Probability of Saturated Load Status

The load status of a sensor node at any slot is correlated with previously arrived data packets. If a previously arrived data packet has been either transmitted or dropped, the load status of sensor node at the current slot is unsaturated. Otherwise, the sensor node is in saturated load status. Considering the data packet inter-arrival time *T*, by excluding the expected time for CCA operation and transmission, the maximum number of slots for current data packet’s backoff operation without affecting next data packet’s transmission can be estimated as:

(22) $$T_{bf}=T-(\sum_{i=1}^{NB_{\max}}{\left(1-p_{cca}^{s}\right)}^{i-1}\Delta_{cca}+\Delta_{L}),$$

where $\Delta_{cca}$ is the expected channel sense duration and $\Delta_{L}$ is the estimated time of sending each data packet in average:

(23) $$\Delta_{cca}=1+P_{cca1}^{s}=\frac{1+LP_{cca}^{s}}{1+L},$$

(24) $$\Delta_{L}=\left[1-{\left(1-P_{cca}^{s}\right)}^{NB_{\max}}\right]\cdot L,$$

Hence, the probability of sensor node in unsaturated load status is:

(25) $$P_{unSAT}^{}=F_{}(x\leq T_{bf}),$$

Similarly, the probability of sensor node in saturated load status can be expressed as:

(26) $$P_{SAT}^{}=1-F_{}(x\leq T_{bf}),$$

#### 4.2.3. The Analysis of Single Node’s CCA1 Operation

Considering the successful probability of CCA1 operation, the expected number of CCA1 operations for one data packet is $\sum_{i=1}^{NB_{\max}}{\left(1-p_{cca}^{s}\right)}^{i-1}$. Whether a node is in saturated load status or not, the expected time for one data packet’s transmission attempt can be expressed as:

(27) $$T_{pkt}=\sum_{k=0}^{N_{NB}-1}f(k)\cdot k+(\sum_{i=1}^{NB_{\max}}{\left(1-p_{cca}^{s}\right)}^{i-1}\Delta_{cca}+\Delta_{L}),$$

so the probability of sensor node performing CCA1 operation within each data packet’s transmission attempt period should be:

(28) $$\omega_{}^{1}=\frac{\sum_{i=1}^{NB_{\max}}{\left(1-p_{cca}^{s}\right)}^{i-1}}{T_{pkt}},$$

It should be mentioned that if one sensor node finished current data packet’s transmission attempt and no other data packet need to be transmitted, the node will not perform a CCA operation until the next data packet comes.

### 4.3. Channel-Node Combined Analysis

#### 4.3.1. The Probability of CCA1 Operation

With the combination of (5) and (28), the probability of CCA1 operation in channel in any given slot *t* is:

(29) $$\tau_{}^{1}(t)=1-{\left(1-\omega_{}^{1}\right)}^{M(t)},$$

The term $M\left(t\right)$ stands for the number of active sensor nodes at slot *t*, which can be estimated as:

(30) $$M\left(t\right)=\frac{2T_{pkt}}{T}M,$$

We can observe that the value of $\tau_{}^{1}\left(t\right)$ varies along time, as does to the value of $P_{cca}^{S}$.

According to (12), (20) and (22), we can find that with certain values of *N*, *T*, *L* and backoff parameters, $\tau_{}^{1}\left(t\right)$ can be expressed as a function of $P_{cca}^{S}$:

(31) $$\tau_{}^{1}=G(p_{cca}^{s}),$$

With the combination of (5) and (31), both $\tau_{}^{1}\left(t\right)$ and $P_{cca}^{S}$ can be carried out. We can find that in any given network with specified values of *N*, *T*, *L* and backoff parameters, both $\tau_{}^{1}\left(t\right)$ and $P_{cca}^{S}$ can be estimated analytically.

#### 4.3.2. The Probability of Collision

Collisions happens when more than one node successfully accesses the same channel simultaneously. Besides, channel capacity can be taken as a motivating factor of data packet collision. The maximum average number of data packets that one node can transmit within one contention period can be calculated as:

(32) $$n_{K,L}^{\max}=\left\lceil \frac{K-1}{\left(L+2\right)\cdot M}\right\rceil ,$$

If a sensor node successful accesses the channel more than $n_{K,L}^{\max}$ times, more data packets will suffer collisions. Another limitation of single node’s successful channel access is the number of data packet that arrives within one contention period. It is rational that the number of arrived data packet should no less than the number of successful channel access within a given period. By taking channel capacity and data packet arrival rate into consideration, we proposed a correction factor χ:

(33) $$\chi =\left\{\begin{array}{ll} \frac{\min(n_{K,L}^{\max},\frac{1}{T})}{\omega^{1}\cdot P_{cca}^{S}\cdot \left(K-1\right)} & ,\omega^{1}\cdot P_{cca}^{S}\cdot \left(K-1\right)>\min(n_{K,L}^{\max},\frac{1}{I})\\ 1 & ,\omega^{1}\cdot P_{cca}^{S}\cdot \left(K-1\right)<=\min(n_{K,L}^{\max},\frac{1}{I}) \end{array}\right.,$$

The probability of data packet collision can be expressed as:

(34) $$P_{colli}=1-\chi \cdot {\left(1-\omega^{1}\right)}^{M-1},$$

## 5. Performance Metrics

### 5.1. Throughput Analysis

Packets are unsuccessfully received due to two reasons: channel access failure and packet collision. The probability of successful transmitting one data packet can be expressed as follows:

(35) $$P_{}^{S}=\omega_{}^{1}\cdot P_{cca}^{S}(1-P_{colli}),$$

For a channel shared by *M* sensor nodes, the number of data packets successfully transmitted within one contention period can be calculated as:

(36) $$\varphi =M\cdot K\cdot \omega_{}^{1}\cdot P_{cca}^{S}(1-P_{colli}),$$

### 5.2. Energy Consumption Analysis

Since the coordinator is always in receiving mode through the contention period, we shall only focus on sensor nodes’ energy consumption. Specifically, we study the average energy consumptions of single node in one slot. As mentioned before, a sensor node has four modes, hence, the energy consumption consists of four parts.

As is derived earlier, the expected number of slots that a single node spends on CCA1 and CCA2 operations in one contention period can be expressed as:

(37) $$K_{CCA}^{E}=K\omega_{}^{1}\left(1+P_{cca1}^{S}\right),$$

Hence, the energy that a single node consumes for channel sense in one contention period is:

(38) $$E_{CCA}^{E}=K_{CCA}^{E}\cdot \varepsilon_{RX}^{},$$

where $\varepsilon_{RX}^{}$ stands for the energy that one node needs for channel sensing in one slot.

Once a sensor node accesses the channel successfully, its transmission starts at the next slot and then lasts for *L* slots, so the expected number of slots that a single node spends for transmission in one contention period can be expressed as:

(39) $$K_{TX}^{E}=K\omega^{1}P_{cca}^{S}L,$$

Accordingly, the energy that a single node consumes in one contention period for transmission is:

(40) $$E_{TX}^{E}=K_{TX}^{E}\cdot \varepsilon_{TX}^{},$$

where $\varepsilon_{TX}^{}$ stands for the energy that node needs for transmission in one slot.

Based on previous analysis, the expected time that one node spends for sleeping in one contention period can be expressed as:

(41) $$K_{SLP}^{E}=n_{S}X_{idle},$$

With the energy that a node in sleep mode consumes in one slot, $\varepsilon_{SLP}^{}$, we can calculate the average energy that one node consumes for sleeping in one contention period as:

(42) $$E_{SLP}^{E}=K_{SLP}^{E}\cdot \varepsilon_{SLP}^{},$$

The time that one node spends for backoff in one contention period can be expressed as:

(43) $$K_{BF}^{E}=K-K_{CCA}^{E}-K_{TX}^{E}-K_{SLP}^{E},$$

Its average energy consumption is:

(44) $$E_{BF}^{E}=K_{BF}^{E}\cdot \varepsilon_{BF}^{},$$

Hence the average energy that one node consumes in one slot can be expressed as:

(45) $$e=\frac{1}{K}\left(K_{CCA}^{E}\cdot \varepsilon_{CCA}^{}+K_{TX}^{E}\cdot \varepsilon_{TX}^{}+K_{SLP}^{E}\cdot \varepsilon_{SLP}^{}+K_{BF}^{E}\cdot \varepsilon_{BF}^{}\right),$$

## 6. Simulation and Numerical Results

### 6.1. Simulation Setup

In the following experiments, we assume that all nodes are communicating with a maximum bit rate of 250 kbps. The value of *BO* and *SO* are equal to 6, which means only CAP exists in each *BI* and the number of slots *K* in each CAP is 1536. Unless specified, all the nodes’ parameter settings are with default values. Besides, each sensor node is assumed to generate data packets with a fixed period of *T* slots. All the data packets are of the same length L=8 (in terms of slots). Since Cao et al. took Mica2 as their reference platform in [17], to present a straightforward comparison with previous researches, we also take Mica2 as reference in the following simulation. It should be noted that more recent reference platforms, such as MicaZ, TelosB and TelosB-clones are also compatible with the proposed model. All the simulation results we present are the mean values of 10^4^ independent simulation runs for each parameter setting. Those results are presented in Table 2.

### 6.2. Model Validation

In this section, the network scale is fixed to 20 nodes. By changing the packet inter-arrival time from 50 slots to 500 slots, a wide range of traffic loads are simulated. In the following comparison, we take the OMNeT++ simulation programs proposed by Kirsche et al. in [22] as the real scenario reference. The simulation programs have been verified in their research have been referenced in several studies [22,23,24]. Besides, in order to present the different characteristics of periodic traffic from unsaturated traffic and saturated traffic, two more analytical models targeting unsaturated traffic [3] and saturated traffic [5] are also taken into comparison. The results of the comparison are presented and analyzed from two perspectives, local statistics and overall statistics.

**Figure 5:**
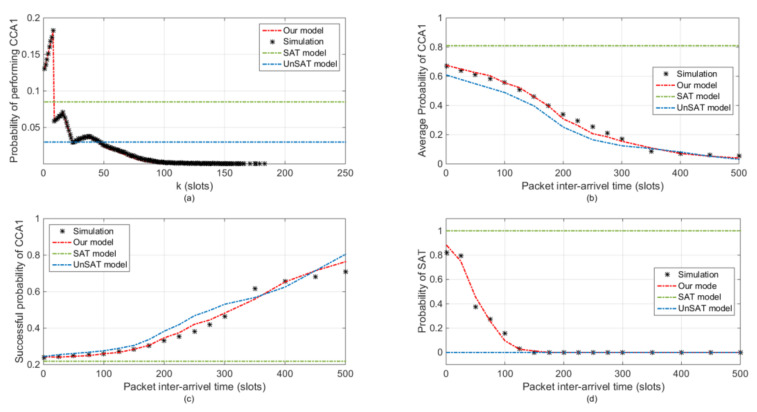
Local statistics comparison. (**a**) Probability of performing CCA1 $\omega^{1}$. (**b**) CCA1 occurrence probability $\tau_{}^{1}$. (**c**) Successful CCA1 probability $P_{cca}^{S}$. (**d**) Saturated load statues probability $P_{SAT}^{}$.

Figure 5 presents the comparison of local statistics, which includes the performance of the MAC layer and the load status of a single node. As shown in Figure 5a, the prediction of SAT and UnSAT models are independent of time, since they pursue stationary statistics of the MAC performance. In contrast, our model accurately predicts the evolutions of $\omega^{1}$ along time. As shown in Figure 5a, after the first backoff which expires with equal probabilities in the first eight slots, a node which experiences unsuccessful CCA(s) will perform another backoff, which expires with probabilities equally distributed in the future slots. Continuing in this way, we can predict that $\omega^{1}$ first increases during the first eight slots. The peak value is reached at the 7th slot, from which a quick drop takes place. The reason is two-fold: the expiration probability of a newly performed backoff decreases as the backoff exponent increases; and the probability of a node turning to sleep increases since it may have already transmitted its packet. Basically, $\omega^{1}$ will ultimately decrease to 0, providing that the contention period is sufficiently long.

Figure 5b,c show the occurrence and successful probability of CCA1 operation in the channel. The results presented in Figure 5b,c can be explained coherently. According to the channel access mechanism, the occurrences of CCA1 in the channel can only be caused by two reasons, the need for data packet transmission and the failure of the previous channel access attempt. For each node, when its packet inter-arrival time is short, more data packets cause more CCA1 operations. For the entire network, intensive channel access attempts reduce the successful probability of channel access, which further stimulates more CCA1 operations. On the contrary, longer packet inter-arrival time and higher successful channel access probability bring on less CCA1 operations. As shown in Figure 5b, according to the simulation result, the probability of CCA1 operation occurs in the channel decreases as the packet arrival interval increases. However, the successful probability of CCA1 operation keeps increasing as the packet arrival interval increases in Figure 5c. With the assumption of saturated load status, all nodes in the SAT model always have packets to be transmitted. Hence, despite the changes in the packet inter-arrival time, both the occurrence and successful probability of CCA1 operation keep constant in the SAT model. Since short packet inter-arrival time makes the load status approach to saturation, the performance difference between SAT model and simulation result becomes larger as packet inter-arrival time increases. In the network with larger packet inter-arrival time, the UnSAT model can obtain accurate channel access operation results. However, the simulation result of UnSAT model in the scenarios with short packet inter-arrival time is not accurate. This is because the UnSAT model assumes there always exists idle slots before new data packet comes. Owing to the consideration of both channel access duration and packet inter-arrival times, the proposed analytical model fits simulation results very well despite of the packet inter-arrival time.

Figure 5d presents the local performance in terms of the node’s load status. The probability of SAT is estimated by the probability that whether the packet queue of a sensor node is empty when a newly generated packet arrives. As indicated by the curve, we can find out that with large packet arrival interval, node’s load status tends to be unsaturated. Limited by assumptions, in the SAT model, the probability of SAT is always 1. Similarly, the probability of SAT in the UnSAT model is always 0. The proposed model can accurately estimate the load status of single node along with different packet inter-arrival time.

**Figure 6:**
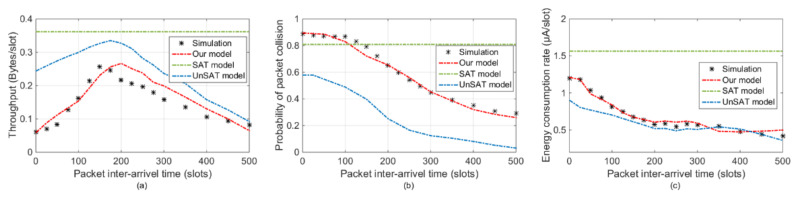
Overall statistics comparisons. (**a**) Aggregate throughput S. (**b**) Packet collision probability $P_{colli}$.(**c**) Energy consumption e.

The overall statistics include the aggregate throughput of one contention period, packet collision probability, and average energy consumption per slot. According to the simulation result in Figure 6a, the throughput of the whole network increases at first and then starts to decrease as the packet inter-arrival time increases. The turning point occurs at around 150th slots. With the default values of channel access parameters, the number of slots that one sensor node spends on one data packet is 133. If the packet inter-arrival time is less than 133, sensor nodes still have the probability of continuous data packet transmission. Hence, shorter packet arrival interval brings more channel access failure and more packet collision, which finally decrease the aggregated network throughput. If the packet inter-arrival time is more than 150 slots, although less channel access failure and packet collision occur with long packet arrival intervals, the total number of generated data packets in a given period also decreases, which leads to a decrement of aggregate throughput. Taking the simulation results as a reference, the proposed analytical model can better predict the aggregate throughput under different packet inter-arrival time than SAT and UnSAT. The estimated throughput of the SAT model is constant and always higher than the simulation results. This is mainly because the assumption of there always have packet to be sent. Although the UnSAT model can characterize the variation of throughput, the estimated value is always higher than the simulation result. It is because UnSAT obtains lower packet collision probability, as is shown in Figure 6b.

As is shown in Figure 6b, the probability of packet collision in SAT model stands still along with packet inter-arrival time changes. The difference between the UnSAT model and the simulation result is consistently obvious. It is because the UnSAT model doesn’t take the limitation of channel capacity and data packet arrival rate into consideration. Compared with the SAT model and UnSAT model, the proposed analytical model fits simulation model very well with different packet arrival intervals, as a result of our comprehensive model design.

Figure 6c shows the average energy consumption of each node. Since the nodes in the SAT model always have packets to send, the energy consumption is constant and always higher than the OMNET++ result. As UnSAT assuming idle slots always exists in contention period, the energy consumption of UnSAT is always lower than OMNET++ result. The difference between UnSAT and OMNET++ simulation is larger with shorter packet arrival interval. Thus, the difference between the proposed model and OMNET++ simulation results is always the smallest.

### 6.3. Single CCA vs. Double CCA

In many analytical models and parameter tuning algorithms for IEEE 802.15.4 MAC layer, SS mode is usually assumed instead of DS mode. Accordingly, to learn about their characteristics, their performance has also been compared in some studies [5,17]. In a saturated traffic scenario, the study of [5] finds that DS mode can provide nodes with higher reliability than SS mode does on different network scales. However, the network throughput of DS mode is always about 10% lower than that of the SS mode. Besides, the average service time for one data packet of SS mode is always lower than the DS mode. The study of [17] compares the SS mode and the DS mode in a periodic traffic scenario. In the simulation of [17], ACK packet and retransmission are both enabled. According to the result, the DS mode outperforms the SS mode on all the considered network scenarios. It is mainly because the node with the SS mode is more easily to transmit packets and thus makes the packet collision probability higher. Apart from the common reason, [17] considers data packets may also collide with the ACK packets in the SS mode. Since an ACK packet is required to be sent with one slot’s gap after a successful transmission in the standard. Nodes with the SS mode may check the channel state in the gap and transmit their data packets right after the gap. This case will not happen with the node in the DS mode.

However, in standard beacon-enabled WSNs, retransmission mechanism and the ACK packet are disabled. Accordingly, the performance of SS mode and DS mode in standard beacon-enabled WSNs may be different. Hence, the following simulation is designed to compare the performance of SS mode and DS mode in standard beacon-enabled WSNs with periodic traffic.

With the following modifications, the proposed model can work in SS mode. Equation (3) should be taken as the function of successful channel access probability:

(46) $$P_{cca}^{S}=\frac{1+\tau_{i}^{1}}{1+\tau_{i}^{1}(1+L)},$$

Equation (37) should be rewritten as:

(47) $$K_{CCA}^{E}=K\omega_{}^{1},$$

The value of expected channel sense duration $\Delta_{cca}$ in (23) should be set to 1. In (32), the term of *L* + 2 should be replaced by *L* + 1.

In this section, the packet inter-arrival time is fixed to 150 slots. By changing the network scale from five nodes to 50 nodes, the performance of the DS mode and the SS mode are evaluated under different traffic loads.

**Figure 7:**
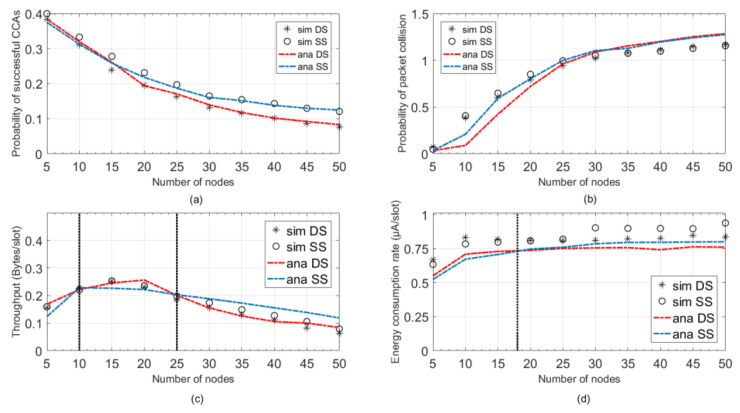
Comparisons between DS mode and SS mode. (**a**) Channel successful access probability $P_{CA}^{S}$. (**b**) Packet collision probability $P_{colli}$. (**c**) Aggregate throughput S. (**d**) Energy consumption e.

Figure 7 presents the comparison result from the local perspective and overall perspective. As is shown in Figure 7a, the successful channel access probability $P_{CA}^{S}$ in DS mode is always lower than SS mode. The difference between the two modes is getting more and more obvious as the number of node increases. To confirm the idle state of a channel, the DS mode requires two successive CCA operations, while the SS mode only needs one. Given the same simulation scenario, the SS mode is more likely to confirm the channel state to be idle. When the network scale is small, since the competition in a channel is not very intensive, nodes can easily find the channel in the idle state. Therefore, the difference between SS mode and DS mode in channel access is not obvious. In a large-scale network, with many nodes need to send data packet, the difference between SS mode and DS mode in channel access starts to turn up. Figure 7b presents the packet collision probability $P_{colli}$ of two modes. We can observe that with fewer nodes in the network, the packet collision probability of two modes is close, which has similar reason as before. As the number of nodes increases, owning to longer channel sensing duration, DS mode can provide a lower packet collision probability than SS mode does. However, with the further increase of node number, a progressive convergence of packet collision probability appears. Limited by the channel capacity, further increase of transmission can bring a network with more frequent and more serve packet collision (e.g., three and more packets’ collision).

From the overall perspective, Figure 7c presents the average aggregate throughput S. We can observe that in a network with less than 10 nodes, the aggregate throughput of two modes is very close, which indicates the performances of SS mode and DS mode in terms of channel access and collision avoidance are very close. While as the scale of network increases, DS mode can maintain higher throughput than SS mode does. It is mainly because nodes in DS mode can prevent more packet collision from happening. If the number of nodes is greater than 25, the aggregate throughput of SS modes starts to overtake the DS mode. Since compared with DS mode, nodes in SS mode spend less time on CCA operations, which slightly decrease the average time spending on a data packet. Hence, the maximum number of data packets that one node can transmit within one contention period is improved. In small scale network, although the performance of packet transmission is close, nodes in DS mode will consume more energy on channel sensing attempt. As is shown in Figure 7d, when the number of nodes is less than 18, the average energy consumption of DS mode is slightly higher than SS mode. However, as the number of nodes increases, the average energy consumption of SS mode surpasses DS mode. Since nodes in SS mode spend much more energy on data packet transmission than DS mode does.

### 6.4. An Estimation of Average Transmission Latency

According to the studies reported in [25,26], the sum of independent non-identically distributed uniform random variables can reasonably be approximated to a normal distribution. Similarly, the distribution of backoff time for one data packet can also be approximated as a normal distribution $N\left(\mu ,\sigma \right)$ and its mean and standard deviation can be calculated as follows:

(48) $$\mu =\sum_{i=1}^{NB_{\max}}\frac{\left(2^{BE_{i}}-1\right)}{2}{\left(1-p_{cca}^{s}\right)}^{i-1},$$

(49) $$\sigma =\sqrt{\sum_{i=1}^{NB_{\max}}\frac{{\left(2^{BE_{i}}-1\right)}^{2}}{12}{\left(1-p_{cca}^{s}\right)}^{i-1}}.$$

Hence, the value of average transmission delay can be approximated as:

(50) $${\tilde{T}}_{pkt}=\mu +(\sum_{i=1}^{NB_{\max}}{\left(1-p_{cca}^{s}\right)}^{i-1}\Delta_{cca}+\Delta_{L}).$$

According to whether the average packet transmission latency is lower than the packet inter-arrival time or not, we can classify networks into two kinds, i.e., delay-bounded networks and delay- unbounded networks. In delay-bounded networks, most data packets will be transmitted or dropped before the next data packet comes. On the contrary, most data packets in delay-unbounded networks have to wait a period of time before been serviced. It can be expected that in delay-unbounded network, the average transmission latency keeps increasing along time. With the estimation of average transmission delay ${\tilde{T}}_{pkt}$, we can confirm whether a network with given network load settings and specified channel access parameters is delay bounded or not.

**Figure 8:**
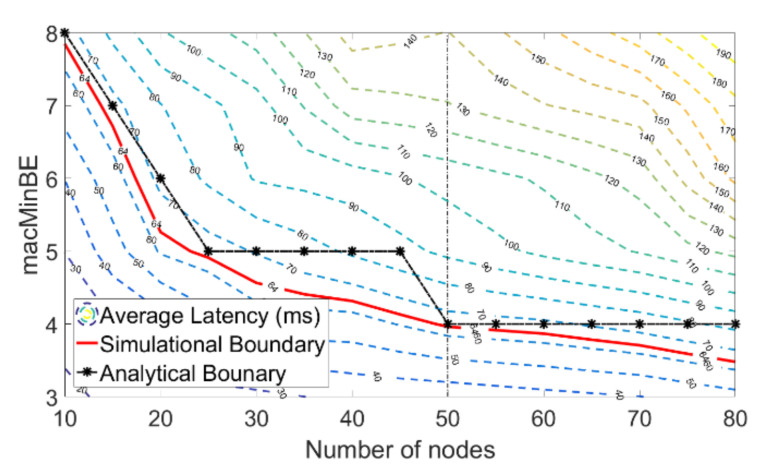
Contour plot for average transmission latency versus network scale and *macMinBE* given PI = 200 slots.

**Figure 9:**
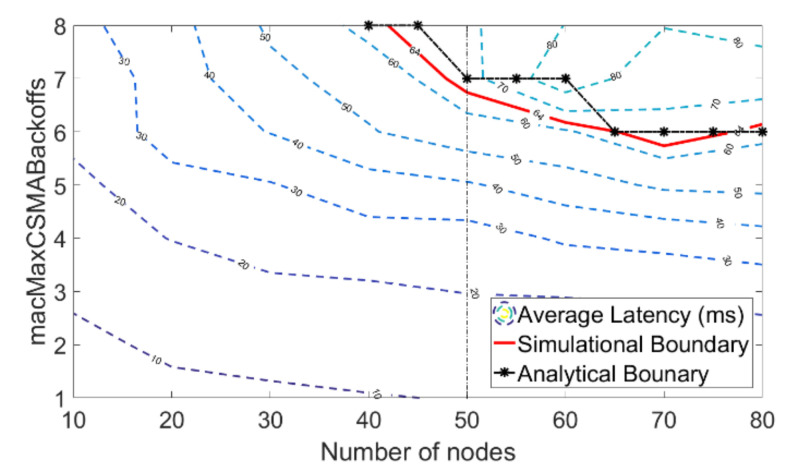
Contour plot for average transmission latency versus network scale and *macMaxCSMABackoffs* given PI = 200 slots.

In the following experiments, packet inter-arrival time *T* is fixed to 200 slots, i.e., 64 ms. The scale of the network ranges from 10 nodes to 80 nodes. Under each network scales, different values of *macMinBE* and *macMaxCSMABackoffs* are simulated. Figure 8 presents the simulation results with different *macMinBE* values under different network scales. The contours present the average transmission delay. In both figures, the contour interval is equal to 10 ms. The red curve stands for the boundary according to simulation results and the analytical boundary is presented as a blacked curve with black asterisks. Similarly, Figure 9 resets the simulation results with different *macMaxCSMABackoffs* values under different network scales.

Compared with Figure 9, the contour of average transmission latency in Figure 8 is denser, which indicates the average transmission latency has fluctuated greatly. Taking the network with 50 nodes as an example. According to Figure 8, the average transmission latency ranges from less than 40 ms to 140 ms. However, the maximum average transmission latency is less than 70 ms in Figure 9. It means *macMinBE* has more influence on transmission latency than *macMaxCSMABackoffs*. In any given network with fixed network scales, increasing the value of *macMinBE* can significantly increases the average packet transmission latency, which probably changes the network from delay- unbounded to delay-unbounded.

What’s more, both Figure 8 and Figure 9 also present the boundaries between delay-bounded networks and delay-unbounded networks. Based on the simulation results, we take the curve that average transmission latency equals to packet inter-arrival time *T* as the simulational boundary. The analytical boundary is obtained by comparing ${\tilde{T}}_{pkt}$ with *T*. We can find out that the analytical boundary always lies above and right of the actual boundary, which means the estimated average transmission latency $T_{pkt}^{\prime }$ is always less than the actual average transmission latency $T_{pkt}^{}$. It is mainly because the estimated average backoff time μ is always less than the actual average backoff time. It is because the value of backoff is always larger than 0, while the value of the independent variable μ in the normal approximation is not. Hence, with this approximation, we can conduct a conservative estimation of whether a specified network’s average transmission latency is bounded by its packet inter-arrival time or not.

## 7. Conclusions

In this paper, we firstly proposed an adequate analytical framework to accurately characterize the MAC behavior of WSN nodes with periodic traffic patterns. The framework is unfolded from two perspectives, channel state and node state. From the perspective of channel state, the relationship between the occurrence of CCA1 in channel $\tau_{}^{1}$ and its successful probability $p_{cca}^{s}$ was formulated through a Markov chain-based channel state model. From the perspective of node state, the distribution of a single node’s backoff time was carried out with the help of discrete time signal processing analysis. Then, we deepened the analysis by translating the interaction among key parameters, the probability of a single node’s CCA1 operation in a slot $\omega_{}^{1}$ and successful channel access probability $p_{cca}^{s}$ into two parametric relationships. By solving these two relationships simultaneously, we can estimate the occurrences probability of CCA1 ($\omega_{}^{1}$ and $\tau_{}^{1}$) and the channel access probability $p_{cca}^{s}$ with given network scale, data packet’s inter-arrival time and channel access parameters. Based on the analytical framework, key performance statistics of single node and whole network were also formulated. With extensive simulations, the proposed framework is validated to be accurate and scalable. Afterward, the performance of SS mode was analyzed and compared with the DS mode. According to the comparison result under different network scenarios, applicable network scenarios of two modes were demonstrated respectively. Finally, with the normal approximation of backoff distribution, a conservative estimation of data packet’s average transmission latency was carried out.

## Figures and Tables

**Table 1:** Basic notations.

Notation	Values	Description
*M*	Default: 2	Number of sensor nodes in network
*K*	Default: 1536	Number of slots in one contention period
*T*	Default: 250	The inter-arrival time of data packet in slots
*L*	Default: 8	The length of one data packet in slots
*macMaxCSMABackoffs*	Range: 0–5 Default: 4	Maximum number of backoff stages
*macMaxBE*	Range: 3–8 Default: 5	Maximum backoff window exponent
*macMinBE*	Range: 0–7 Default: 3	Minimum backoff window exponent permeability
*NB*	Range: 0–*macMaxCSMABackoffs*	Number of backoff times
*BE*	Range: *macMinBE*–*macMaxBE*	Backoff exponent
*CW*	Default: 2	The length of Contention Window

**Table 2:** Power consumption of Mica2 in different states [17].

Modes	Energy Consumption
Transmitting $\varepsilon_{TX}^{}$	24.6 mA
Receiving $\varepsilon_{RX}^{}$	17.2 mA
Idle $\varepsilon_{BF}^{}$	1.617 mA
Sleep $\varepsilon_{SLP}^{}$	0.297 mA
